# Asthma in Patients With Confirmed Pulmonary Sarcoidosis

**DOI:** 10.1155/carj/7829487

**Published:** 2026-01-28

**Authors:** Agata Anna Lewandowska, Dorota Waśniowska, Małgorzata Kołodziej, Helena Mirus-Arabik, Aleksandra Gaczkowska, Ola Duszyńska, Krzysztof Bułat, Michał Graczyk, Cezary Rybacki

**Affiliations:** ^1^ Clinical Department of Pulmonology, Allergology and Pulmonary Oncology, 10th Military Clinical Hospital with Polyclinic, Bydgoszcz, Poland; ^2^ Department of Palliative Care, Collegium Medicum in Bydgoszcz, Nicolaus Copernicus University, Toruń, Poland, umk.pl; ^3^ Faculty of Medicine, Bydgoszcz University of Science and Technology, Bydgoszcz, Poland

**Keywords:** asthma, bronchial hyperresponsiveness, bronchial provocation challenge, methacholine, sarcoidosis

## Abstract

Pulmonary sarcoidosis and asthma can present identical symptoms, making clinical evaluation difficult if the two diseases overlap. Diagnostic challenges often lead to either overdiagnosis of asthma in patients with confirmed sarcoidosis or withholding appropriate asthma treatment. The true prevalence of patients with bronchial hyperresponsiveness, the hallmark of asthma, among sarcoidosis patients remains unknown, although it is suspected to be significantly higher compared to the general population. Therefore, a positive bronchial challenge test, often considered crucial for confirming asthma in symptomatic individuals with normal spirometry, should not be regarded as decisive in patients with pulmonary sarcoidosis. However, the coexistence of both diseases is possible and should always be considered. Caution is advised as patients prematurely and incorrectly diagnosed with asthma are exposed to unnecessary medical costs and lifelong treatment. Nevertheless, inhaled glucocorticosteroids and bronchodilators may be temporarily used in sarcoidosis patients because of their beneficial effect on symptom control, regardless of a concurrent asthma diagnosis. Despite the existing evidence that patients with sarcoidosis can develop asthma and atopy, the complex cellular pathways and genetic predispositions involved in the pathogenesis of both diseases remain largely unknown. To address these issues in clinical practice, the article aims to discuss the mechanisms involved in the etiopathogenesis of asthma and sarcoidosis and to analyze the available diagnostic and therapeutic methods relevant to both conditions.

## 1. Introduction

Sarcoidosis is a heterogeneous disease of unknown etiology with histopathological features of non‐necrotizing granulomas, predominantly involving lungs and intrathoracic lymph nodes in approximately 90% of patients [1–3]. The incidence of sarcoidosis varies between 1 and 15 per 100.000 inhabitants, depending on the region [4]. Sarcoidosis starts in adults between 25 and 40 years old in approximately 70% of cases, in all racial and ethnic groups, with a second peak in 50‐year‐old women and a strong predilection for black populations [5–7].

The most common symptoms of pulmonary sarcoidosis, described in over 50% of patients, include dry cough, shortness of breath, and chest pain [8]. In 30% of cases, patients experience fever, weight loss, chronic fatigue, and other symptoms if extrapulmonary manifestation is present (15%–25% of cases) [8]. Sarcoidosis is characterized by a very heterogeneous course, which in two‐thirds of patients leads to spontaneous remission within 12–36 months [5]. The mortality rate is less than 10%, with the most fatal cases concerning patients suffering from advanced lung disease and cardiac complications [2]. The acute onset is associated with more favorable prognosis, as in Löfgren syndrome, characterized by symptoms such as fever, hilar lymphadenopathy, erythema nodosum, and arthritis [2]. In the remaining patients, the disease takes a chronic course, and in the presence of cardiac, neurologic, renal, and fibrotic pulmonary involvement, associated with higher mortality, systemic treatment is indicated [2, 5].

Asthma is a heterogenous disease characterized by chronic airway inflammation [9, 10]. It affects more than 300 million people worldwide of all ethnic groups and ages with the incidence estimated to be 1%–29% in different countries [9, 11–13]. Cough, wheezing, shortness of breath, and chest tightness are considered the most common symptoms among asthma patients [10], which together with expiratory airflow limitation vary in time and intensity [9, 14]. Based on demographic, clinical, and pathophysiological characteristics, several phenotypes are distinguished, such as allergic asthma, nonallergic asthma, late‐onset asthma, asthma with persistent airflow limitation, and asthma associated with obesity [9]. It is listed as one of the most common comorbidities encountered in sarcoidosis, among chronic obstructive pulmonary disease (COPD), hyperlipidemia, obesity, thyroid disease, diabetes, osteoporosis, hypertension, chronic renal disease, or coronary heart disease [6].

Frequent delays and diagnostic uncertainty are common in the case of sarcoidosis [15]. The situation becomes even more complicated in patients with confirmed sarcoidosis and accompanying bronchospastic/asthmatic symptoms, who present obstructive ventilatory defect and/or bronchial hyperresponsiveness (BHR) documented in pulmonary function testing. Failure to diagnose asthma in sarcoidosis patients, and therefore withholding proper treatment, affects the patients’ quality of life. In addition, it is unclear whether patients with BHR in sarcoidosis should be treated similarly to patients with asthma. The purpose is to highlight the clinical problem, arising from the symptom similarities of asthma and pulmonary sarcoidosis in some patients, as well as investigate the suspected extent of coexistence of both diseases. In an attempt to identify potential research directions, the article aims to discuss the mechanisms involved in the etiopathogenesis of asthma and sarcoidosis and to analyze the available diagnostic and therapeutic methods in the course of both diseases based on current literature.

## 2. Methodology

The review was conducted using literature available in PubMed, Scopus, and Web of Science medical databases. A thematic analysis was based on the relevant articles published between 1985 and 2025. The keywords used to perform the search included the following: “asthma”, “sarcoidosis”, “bronchial hyperresponsiveness”, “bronchial provocation challenge”, and “methacholine”. The review encompassed review articles, meta‐analyses, systematic reviews, society guidelines, and case reports, as well as prospective and retrospective studies. The search was restricted to articles published in English.

## 3. Etiopathology

The underlying mechanisms of asthma are multifaceted, determined by genetic background and obesity, as well as several environmental and psychosocial factors [9, 11, 16]. The airway obstruction is a result of inflammatory infiltration mediated by activated immune agents, such as dendritic cells, eosinophils, neutrophils, lymphocytes, innate lymphoid cells, and mast cells [10]. Studies indicate that airway epithelium can release cytokines in a response to an injury, infection, or pollutants [11]. In case of asthma, such a reaction leading to airflow limitation can be triggered by exercise, allergen or irritant exposure, change in weather, or respiratory infection [9].

The pathogenesis of allergic asthma is associated with T helper 2 (Th2) cells, a distinct lineage of CD4+ effector T‐cells, and type 2 innate lymphoid cells (ILC2), which after antigen presentation release type 2 cytokines, such as IL‐4, IL‐5, IL‐9, and IL‐13, and therefore lead to excessive influx of eosinophils and immunoglobulin E (IgE) in the airways [10, 11, 17, 18]. ILC2 cells are also activated by IL‐25, IL‐33, and thymic stromal lymphopoietin (TSLP) [18]. The mediators released by mast cells and basophils, including leukotrienes, histamine, and interleukins, irritate smooth muscle cells of the airways and induce bronchoconstriction [11, 18]. The inflammatory mechanisms of type 2 (Th2‐high) asthma are presented in Figure 1 [11].

**Figure FIGURE 1:**
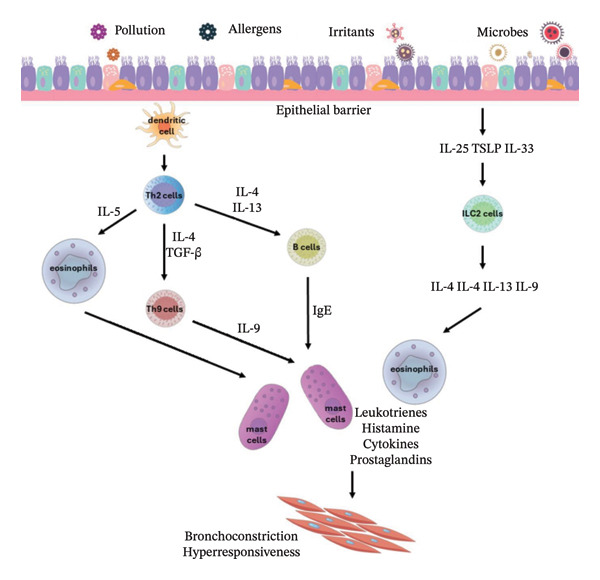
Mechanisms involved in type 2 asthma [11]; thymic stromal lymphopoietin (TSLP), type 2 innate lymphoid cells (ILC2), and immunoglobulin E (IgE).

Non‐type 2 (Th2‐low), non‐allergic asthma phenotype includes neutrophilic asthma and paucigranulocytic asthma, mediated by non‐Th2 cytokines, such as IL‐17, as well as tumor necrosis factor‐α (TNF‐α) and interferon‐γ (IFN‐γ), both activated by Th1 cells [11, 19]. In non‐type 2 asthma, high levels of IL‐17, mostly produced by CD4+ Th17 cells, are found in serum, sputum, and bronchoalveolar lavage (BAL) [11]. As IL‐17 is implicated in asthma development, a full understanding of Th17 cells may be of key importance in the control of asthma pathogenesis [20]. However, new evidence suggests inflammation‐independent processes, involving molecules such as protein kinases, adapter proteins, and others, associated with asthma pathogenesis and progression [11].

In the progression and development of sarcoidosis, studies take into consideration the genetic susceptibility, environmental factors, and dysregulation of the immune system leading to autoimmunity, as well as allergic response induced by unidentified antigens provoking macrophage and T‐cell‐regulated immune reaction followed by the release of cytokines, chemokines, and reactive oxygen species [6, 21, 22]. The etiological agents may include microbes (infectious exposure), as well as organic and nonorganic compounds, often in the form of inhaled bioaerosols, such as musty odors or industrial dust [6, 21]. The immunologic feature of sarcoidosis and granuloma formation shows strong polarization toward Th1 cells with spontaneous release of IL‐2 and IFN‐γ [23]. The immune system responds to undegraded antigens by antigen‐presenting cells, such as dendritic cells, alveolar macrophages, and alveolar epithelial cells, which also increase levels of TNF‐α, IL‐12, IL‐15, IL‐18, macrophage inflammatory protein‐1, monocyte chemoattractant protein‐1, and granulocyte macrophage colony‐stimulating factor [6]. Presentation of the antigens to CD4+ T‐cells, together with the production of Th1/Th17 cytokines, eventually results in sarcoid granuloma formation, comprised of macrophages, epithelioid cells, giant cells, and T‐cells [6, 21]. Simplified inflammatory mechanisms of both non‐type 2 asthma and sarcoidosis are presented in Figure 2 [11, 24, 25].

Figure FIGURE 2Mechanisms involved in non‐type 2 asthma (a) and sarcoidosis (b) [11, 24, 25]; antigen‐presenting cells (APCs).

**Figure:**
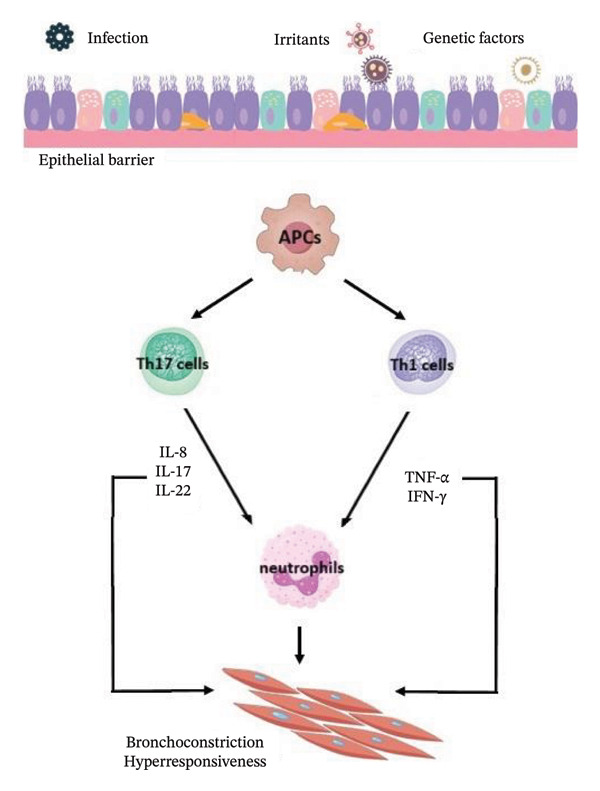
(a)

**Figure:**
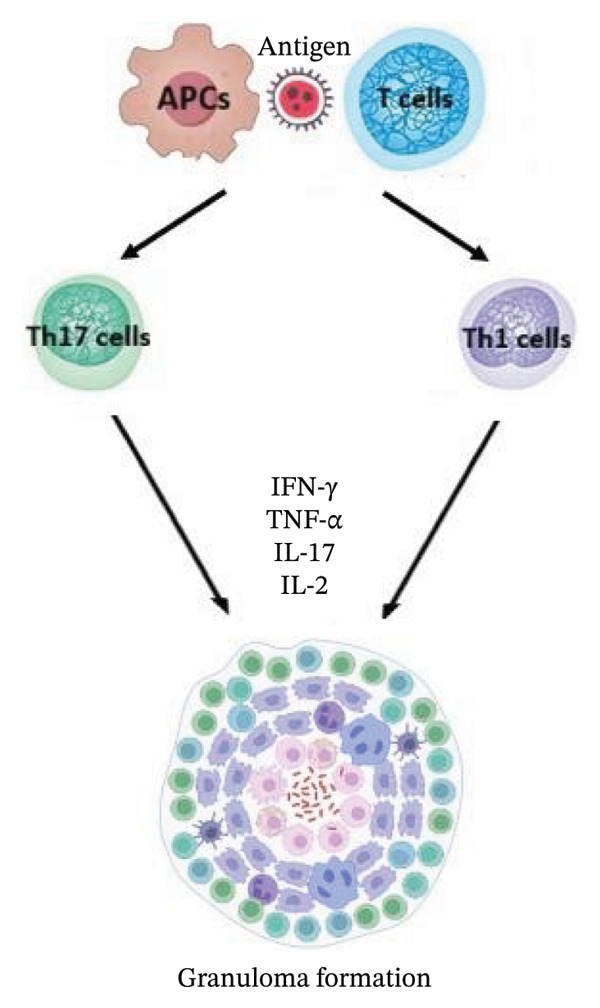
(b)

In the case of active Th1 response, the Th2 cytokine profile (IL‐4, IL‐5, and IL‐10) is suspected to be decreased instead in sarcoidosis patients [23]. In an attempt to counterbalance the proasthmatic effects of Th2 on bronchial hyperreactivity, allergen‐specific Th1 lymphocytes were transferred into naïve mice [20, 26]. Surprisingly, the airway inflammation intensified, together with IFN‐γ production and eosinophil activation [20, 26]. It can be concluded that the Th1/Th2 relation is not as simple as a dichotomous counterbalance, but rather a harmful combination in asthmatic individuals [20, 26]. Studies indicate that Th1 cytokines may contribute to asthma pathogenesis [11]. In a study examining BAL of children with severe asthma, authors demonstrated a dominant Th1 profile and cytokines atypical to allergy in the lower airways, which contraindicates established beliefs and warrants further assessment of Th1 involvement in asthma [27].

Despite the fact that sarcoidosis is characterized by a predominant Th1 type immune response, patients with sarcoidosis can still develop asthma and atopy (associated with Th2 response) with a frequency similar to the healthy population [28, 29]. The role of Th2 is actively studied in the context of progression of sarcoidosis, as the transition from Th1 to Th2 cytokine profile can happen in the chronic course of the disease, perhaps as a response to prolonged inflammation [30, 31]. The involvement of Th2 lymphocytes seems to occur especially in patients with pulmonary fibrosis, associated with the release of cytokines, such as transforming growth factor‐β (TGF‐β), IL‐13, and C‐C motif ligand 2 (CCL2) [30, 32]. IL‐13 mRNA expression is also elevated in sarcoidosis patients, which is a key cytokine of Th2 cells [30]. However, the mechanisms of pulmonary fibrosis in sarcoidosis, ultimately leading to traction bronchiectasis, honeycombing, and architectural distortion [33], are still poorly understood. There are reports indicating that the treatment of sarcoidosis based on TNF‐α antagonists may cause asthma exacerbation due to a suspected disruption of Th1/Th2 balance with a reduction in Th1 cytokines [34]. Older studies from the 20th century suggest the etiological relationship between asthma and sarcoidosis due to the abnormal immunological reaction present in sarcoidosis associated with the activation of B lymphocytes and acceleration of IgE‐mediated response [22]. Therefore, it is suspected that the inflammation of the airways in coexisting asthma and sarcoidosis tends to improve or deteriorate the course of both diseases [35]. It is also possible that sarcoidosis development causes clinical manifestation and exacerbation of the pre‐existing asthma [2, 22].

### 3.1. Genetic Predisposition

Human leukocyte antigen (HLA) alleles are located on chromosome 6p21 and considered the most polymorphic genes associated with immune response and susceptibility to certain diseases [36]. The majority of studies examined Northern and Western Europe populations, pointing to the major histocompatibility complex II class genes, HLA‐DRB1, considering their crucial role in antigen presentation [37]. The development and clinical phenotype of the disease are determined especially by HLA‐DRB1∗01, HLA‐DRB1∗03, HLA‐DRB1∗04, HLA‐DRB1∗14, and HLA‐DRB1∗15 [4, 21], as well as non‐HLA alleles, such as TGF‐β, TNF‐α, and toll‐like receptor 4 [6]. Moreover, several sarcoidosis risk loci are identified to date—BTNL2, HLA‐B, HLA‐DPB1, HLA‐DQB1, ANXA11, IL23R, SH2B3/ATXN2, IL12B, NFKB1/MANBA, FAM177B, chromosome 11q13.1, and RAB23 [6]. It is worth mentioning that the association between HLA alleles and the phenotype of sarcoidosis is not considered universal, but rather ethnicity‐specific [21]. Studies show that the heritability of the disease is an important factor in the risk of developing sarcoidosis and can reach up to 39% [21].

Several chromosomal regions have been associated with the predisposition to asthma, such as 2q, 3p, 5q, 6p21, and 12q23 [36]. Furthermore, specific loci and HLA haplotypes have been discovered to be implicated in susceptibility to asthma—IL18R1, IL33, SMAD3, ORMDL3, IL2RB, HLA‐DQA1 (e.g., ∗0104 and ∗0201), DQB1 (e.g., ∗0603 and ∗0604), and DRB1∗070,101 [36, 38]. The majority of studies prove the impact of HLA class II genes, despite the inconsistency in different types of asthma, which requires further research [38].

HLA genes have a fundamental role in immunity and are considered a risk factor not only in asthma but also other immune diseases, such as allergic rhinitis (HLA‐DQB1, HLA‐B, and HLA‐G), allergic bronchopulmonary aspergillosis (HLA‐DRB1), chronic rhinosinusitis disorders (HLA‐DQB1∗03), rheumatoid arthritis, and atopic dermatitis (HLA‐B7) [31, 38, 39].

Patients with sarcoidosis are also more prone to developing autoantibodies characteristic for various autoimmune diseases compared to controls, which has been demonstrated in a retrospective study performed on 154 individuals [32]. A study on the European population found that 16% of patients diagnosed with sarcoidosis presented at least one immune‐mediated disease, which was 2‐fold higher than in the general population [2, 40]. Similarly, sarcoidosis patients in Taiwan had an increased risk of autoimmunity disorders compared to control subjects, particularly in the form of autoimmune thyroid disease, Sjogren syndrome, and ankylosing spondylitis [2, 41].

Despite the lack of evidence indicating a genetic predisposition to the simultaneous development of both asthma and sarcoidosis, the aforementioned data highlight the importance of taking detailed family history, which can greatly facilitate the accurate diagnosis of both disorders [34].

### 3.2. Bronchial Hyperresponsiveness Testing

BHR is defined as a reaction to direct (methacholine and histamine) or indirect (exercise, cold air, hyperventilation, and mannitol) stimuli [42, 43]. Although BHR and airway inflammation are considered the hallmark features of asthma [44, 45], they may be present in other disorders, such as sarcoidosis, COPD, congestive heart failure, cystic fibrosis, bronchiectasis, and allergic rhinitis [46–49]. Heavy smokers also tend to present a higher incidence of BHR by over 3‐fold [50]. The parameter used in clinical evaluation of the response to the provocative agent is the threshold dose causing bronchial obstruction, measured most commonly by forced expiratory volume in one second (FEV1) decrease [46]. In elderly children and adults, a 20% FEV1 reduction is recommended as a positive indicator of bronchial provocation test [46, 51].

The most widely used is the methacholine inhalation challenge, which leads to the activation of the muscarinic M3‐Gq protein coupled receptors and the inositol trisphosphate pathway, eventually resulting in the release of intracellular Ca2+ and smooth‐muscle contraction [46]. The mechanism resembles the action of endogenous acetylcholine on the M3 muscarinic receptors of the airways [46]. Histamine, on the other hand, acts on the H1 receptor on bronchial smooth muscle cells resulting in bronchoconstriction via the vagal reflex [52]. The indirect stimuli can act through multiple pathways, majorly via the activation of inflammatory agents by mast cells and others [42], eventually leading to the release of mediators such as histamine in predisposed individuals [47].

Asthma is a disease associated with chronic inflammation, which is likely to contribute to BHR [53]. Some studies suggest that the clinical improvement is correlated with the mitigation of BHR; however, in other groups of patients, there is no change in BHR despite the alleviation of symptoms and lung function impairment [54]. It is worth mentioning that the intensity of BHR cannot be used as an index of airway inflammation, as well as the sole presence of inflammatory mediators in the airways is not sufficient to cause BHR [46]. The complexity of the mechanisms involved in the intensity of bronchospasm in individual subjects is still unknown, which significantly reduces clinical relevance [46].

There is no worldwide accepted gold standard for the diagnosis of asthma [54–57]. There is an agreement in clinical management that the methacholine, histamine, or mannitol provocation tests are supplementary diagnostic tools in the diagnosis of asthma in patients with normal spirometry and indicative symptom history, such as wheezing, dyspnea, and cough, especially in cases of allergen exposure, at the workplace or during respiratory infections [46, 55, 58]. The sensitivity and specificity of the methacholine challenge test among 126 asthma patients were estimated at 77% and 96%, respectively [59].

A study involving 304 adults with a history of physician‐diagnosed asthma confirmed a positive methacholine test in 73% of the cases [60]. However, the authors concluded that the methacholine‐negative subgroup either presented false negative results or the diagnoses were incorrect [60]. Another study assessing the prevalence of BHR in 373 possibly asthmatic children presented similar results—indicating that 74% of the participants were hyperresponsive according to the 20% FEV1 reduction [61]. In comparison, the prevalence of BHR in the general population is estimated at 10%–16% of adults [62, 63].

In sarcoidosis, BHR occurs in 5%–83% of patients and is considered a potential reason for airflow limitation, which is reported in 5%–63% of cases [3]. Patients with sarcoidosis and confirmed BHR tend to present slightly worse airway obstruction, lower vital capacity, and more symptoms, such as wheezing and cough [48]. One explanation is that endobronchial lesions in the course of sarcoidosis cause BHR by excessive production of inflammatory mediators and/or alteration in cholinergic mechanisms [34]. However, the mechanisms leading to BHR in sarcoidosis still require further study [3].

Clinical trials indicate that the methacholine inhalation challenge may cause mild side effects in the form of wheezing, cough, pharyngeal itching, hoarseness, sore throat, shortness of breath, or chest tightness but does not lead to any other serious adverse events requiring prolonged treatment [51, 55, 64]. Although a negative provocative test result can exclude concomitant asthma with high probability in patients with sarcoidosis, a positive result does not confirm it. Therefore, given the diagnostic challenges, the provocation test should be performed only when it is expected to offer clinical benefit [46]. The prevalence of BHR in patients with asthma and sarcoidosis requires further research in order to determine the diagnostic reliability of bronchial challenge tests in both groups, as well as assess the impact of BHR on the quality of the patients’ lives.

### 3.3. Differential Diagnosis

The diagnosis of sarcoidosis is based on radiological signs, histologically confirmed noncaseating granulomas in a biopsied lesion, BAL testing, possible systemic involvement, and exclusion of other conditions [4, 65]. The simplest and most reproducible staging system is based on roentgenographic appearance, which distinguishes hilar lymphadenopathy (stage I), hilar lymphadenopathy with parenchymal infiltration (stage II), parenchymal infiltration without hilar lymphadenopathy (stage III), and pulmonary fibrosis (stage IV) [66, 67]. Currently, the cornerstone of pulmonary sarcoidosis diagnosis is computed tomography (CT), both high‐resolution and conventional with intravenous contrast to visualize enlarged lymph nodes [4]. The typical high‐resolution CT images in sarcoidosis feature bilateral hilar/mediastinal lymph node enlargement with perilymphatic micronodules scattered along the bronchovascular bundle, fissures, and pleura, often forming larger conglomerations, or a “galaxy sign”—highly suggestive of pulmonary sarcoidosis [68–71]. Due to the diversity of radiological patterns in sarcoidosis and the need for a more advanced classification, seven nonfibrotic or likely to be fibrotic phenotypes were distinguished in a multinational Delphi consensus study in 2024 to anchor future research [72]. Bronchoscopy with endobronchial/transbronchial lung biopsy and endobronchial ultrasound‐guided lymph node sampling are the main invasive techniques to obtain material for histological evaluation [5]. Figure 3 depicts CT scans of the chest of patients with different patterns of histopathologically confirmed sarcoidosis.

**Figure FIGURE 3:**
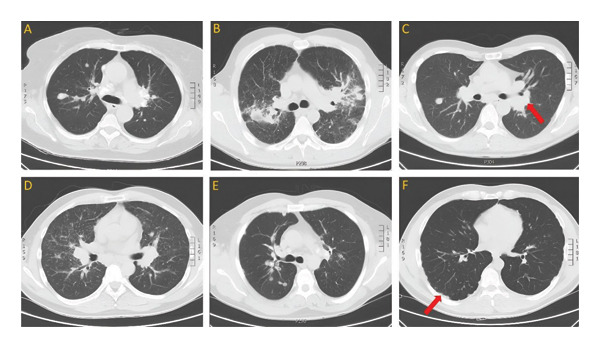
CT scans of the chest of patients with histopathologically confirmed sarcoidosis present hilar lymphadenopathy ((C), marked with an arrow), scattered micronodules (D), and consolidations (B), as well as subpleural (F) and peribronchovascular (A, E) nodules.

In asthma, the diagnosis is based on the clinical presentation of characteristic symptoms and evidence in the form of variable expiratory airflow limitation obtained from bronchodilator reversibility testing, excessive variability during PEF monitoring, variation in FEV1 between visits, a significant increase in FEV1 after inhaled corticosteroids (ICS), or a positive bronchial/exercise challenge test [9, 11, 14]. All of the above support the diagnosis in the absence of pulmonary parenchymal abnormalities [34]. It is worth mentioning that asthma should also be differentiated from COPD, infection, gastroesophageal reflux disease, chronic rhinosinusitis, heart failure, vocal cord dysfunction, and other disorders of the upper airways [73]. The diagnosis is considered certain in a patient with a suggestive symptom history and reversible airflow obstruction; however, normal spirometry does not exclude asthma [74]. In such cases, methacholine bronchoprovocation testing should be considered [60, 75]. In some patients, the airflow obstruction is irreversible in the case of advanced airway‐wall remodeling and mucus retention, leading to further diagnostic challenges [10].

The most common abnormalities of lung function in sarcoidosis include a restrictive ventilatory defect, characterized by the decrease in static lung volumes, together with a reduction in diffusing capacity of the lungs for carbon monoxide (DLCO) and airway obstruction—occurring more common in sarcoidosis than other interstitial lung diseases [76]. Airflow obstruction, defined as decreased FEV1 to forced vital capacity (FVC) ratio in pulmonary function testing, may be a result of stenosis and extrinsic compression of the airways due to pulmonary fibrosis, hilar lymphadenopathy, or diffuse bronchial granulomatosis [5]. In sarcoidosis patients with airway obstructive syndrome, CT patterns can present bronchial distortion/compression, peribronchovascular thickening, and small airway obstruction [77]. It is worth mentioning that air trapping visualized in expiratory high‐resolution CT scans can occur in patients with both pulmonary sarcoidosis and asthma [78–80]. Apart from the aforementioned mechanisms, airflow obstruction in sarcoidosis patients can be caused by bronchospasm in the course of concomitant asthma [81]. Normalization or significant improvement of spirometry after ICS/bronchodilator therapy and careful observation could support such a conclusion [81].

In a group of 830 subjects with established sarcoidosis, the restrictive ventilatory defect was observed in only 7% of participants, despite the parenchymal involvement in approximately three‐quarters of these patients (stages II–IV) [76]. Airflow obstruction was present in 11.7% of the cases—approximately 6% in stage I and 13%–16% in stages II and III [76]. In the group with any ventilatory abnormality in pulmonary function testing, 39.3% presented mixed defects, thus showing a higher incidence of bronchial obstruction, presumably due to the presence of lesions involving both the parenchyma and airways [76]. Authors of another study enrolling 56 subjects obtained similar results, additionally detecting higher incidence of airflow limitation expressed as lowered maximal expiratory flow in one‐third of all patients and approximately 50% in stage III [3]. However, other data in the literature vary significantly due to differences in race, sex, and environmental factors, indicating that only approximately 44%–56% of patients with confirmed pulmonary sarcoidosis exhibit normal lung function [82, 83]. Among patients with ventilation abnormalities, a restrictive phenotype is more probable in Black patients (41% vs. 9%), as well as isolated DLCO reduction (12% vs. 4%), whereas White patients are more likely to develop an obstructive phenotype (17% vs. 9%) [82]. What is more, men exhibit a higher incidence of the obstructive phenotype compared to women, whereas tobacco smokers tend to have a mixed defect or isolated DLCO reduction, compared to nonsmokers [82].

Sarcoidosis can mimic asthma if dyspnea and cough are present, which is observed in 43% of cases [73]. Among several potential causes of sarcoidosis‐associated cough, airway irritation stimulating afferent nerve fibers and mechanical damage secondary to endobronchial granulomatous inflammation are considered the most common [1]. On the other hand, BHR confirmed by methacholine challenge testing generally supports the asthma‐like mechanism [1, 84]. However, the cough etiology may not be related to any of the above or be the result of a primary nerve disorder described as “cough reflex hypersensitivity” [1].

In BAL testing, characteristic finding in sarcoidosis is a lymphocytic alveolitis in 80% of cases, T lymphocyte CD4/CD8 ratio over 3.5 in 50% of cases, and T‐cells expressing the receptor Vα2.3 over 10.5%, as well as CD103+CD4+ T‐cells count [4, 5]. Combining CD4/CD8 and CD103+CD4+/CD4 ratios can be used as a diagnostic tool, showing a specificity of 92% and positive predictive value of 93% [5]. Hypercalciuria, increased serum level of angiotensin‐converting enzyme, and soluble IL‐2 receptor are not considered specific biomarkers of sarcoidosis, although they can support the diagnosis, as well as the initial evaluation and monitoring of organ involvement [4, 85, 86].

Considering the pathological mechanisms, elevated eosinophil, IgE, and periostin serum levels, as well as fractional exhaled nitric oxide and sputum eosinophilia, are practical biomarkers for type 2 asthma, whereas sputum or BAL neutrophils can be used to help the diagnosis of non‐type 2 asthma [11, 34, 87]. Nitric oxide is released by airway epithelium as a result of IL‐13 activity and is considered a marker of Th2 inflammation [11]. However, due to its low specificity, elevation of fractional exhaled nitric oxide has not been established as a useful test for ruling in or out the diagnosis of asthma [9]. Skin prick testing could prove beneficial as the presence of atopy increases the probability of asthma in patients with indicative respiratory symptoms [9, 88, 89].

For both diseases, a definitive test does not exist, and the diagnosis is based on clinical, radiological, and laboratory evidence (Figure 4) [34, 73]. In patients previously diagnosed with pulmonary sarcoidosis, asthma may be impossible to exclude or confirm at single evaluation based on available diagnostic tests [34]. Observed variability in symptom severity and respiratory mechanics tests over time may suggest the coexistence of the two diseases [34]. It is worth mentioning that asthma does not involve pulmonary parenchymal abnormalities, and its exacerbations are not associated with the progression of radiological lesions. Caution is recommended as individuals incorrectly diagnosed with asthma are burdened with the cost and side effects of unnecessary medications [60]. The consequences further involve the misuse of resources, failure to discover the actual pathology behind the patient’s symptoms, and impaired reliability of asthma research [60].

**Figure FIGURE 4:**
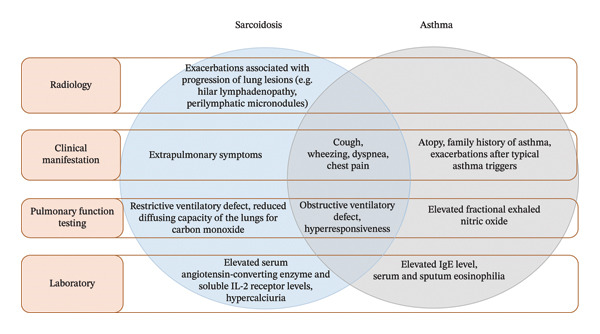
Characteristics of asthma and sarcoidosis in clinical practice [34].

### 3.4. Treatment

ICS are the mainstay in the treatment of asthma, whereas rescue medicines, such as (short‐acting beta‐2 agonists) SABA, are used to rapidly reduce or reverse airflow limitation [14, 90]. Other drugs used in symptom control are long‐acting beta‐2 agonists, muscarinic receptor antagonists, and leukotriene receptor antagonists [91]. Patients tend to overuse SABAs when symptoms worsen, which in asthma is associated with increased risk of death [90]. Therefore, asthmatic patients require stepwise and control‐based management, involving systematic assessment of symptoms, risk factors, and pharmacological treatment [90]. Adequate diagnosis is crucial to manage the treatment and improve the quality of patient’s life, especially in the case of adult‐onset asthma, often more challenging and requiring higher doses of ICS than childhood asthma [13]. Additionally, it is beneficial that oral glucocorticosteroids (OCS), which are the first‐line treatment for sarcoidosis, are also used to control severe asthma symptoms [34, 92, 93].

Considering that sarcoidosis can cause troublesome cough, ICS are sometimes used for symptom relief if OCS, associated with a higher risk of adverse effects, are not indicated and reserved for patients with persistent pulmonary lesions or progressively reduced pulmonary function [94–98]. BHR detected by methacholine provocation challenge additionally supports the asthma‐like mechanism, in which case ICS and bronchodilators might suppress bronchial obstruction syndrome in pulmonary sarcoidosis patients [1, 84]. Fibrosis associated with bronchiectasis is another complication, in which introducing bronchodilators could prove beneficial [99].

Evidence indicates that ICS improves symptom control and lung function, as well as possibly diminish the need for OCS [81, 97, 100–103]. However, the existing meta‐analyses and clinical trials regarding the common use of ICS and bronchodilators are inconclusive and, therefore, official guidelines established to date do not recognize indications for such treatment [94, 104–107]. Evaluating the effectiveness of the GCS, whether oral or inhaled, is further complicated by the frequent tendency of sarcoidosis to be self‐limiting [95].

Considering the crucial role of TNF‐α in the process of sarcoid granuloma formation, TNF‐α inhibitors constitute a third‐line treatment option in patients refractory to conventional treatment, both in pulmonary and extrapulmonary sarcoidosis [108–111]. The aim of the therapy is to limit the interaction between activated macrophages and CD4+ T‐cells, therefore preventing the progression of granulomas [112]. According to scarce available findings, TNF‐α inhibitors lead to a decrease in CD4/CD8 ratio, percentage of CD4+ T‐cells expressing the activation marker CD69, and number of mast cells in pulmonary sarcoidosis patients after 6 months of the therapy [108]. Infliximab is the most studied TNF‐α inhibitor; however, available evidence remains inconclusive and sometimes contradictory [108, 112–114]. There is still controversy to address regarding an optimal dosage, therapy duration, and definition of sarcoidosis remission [108, 109, 115].

Apart from the known TNF‐α inhibitors, such as infliximab, adalimumab, and etanercept, novel potential therapies targeting agents, such as granulocyte‐macrophage colony‐stimulating factor, IL‐18, IL‐6, IL‐1, IL‐17, IL‐12/23, cytotoxic T‐lymphocyte antigen 4, B‐cells, Janus kinase, and phosphodiesterase type 4, are investigated due to their activity in sarcoidosis‐affected tissues [113, 116, 117]. Neuropilin 2 (NRP2), a nontyrosine kinase receptor expressed in mast cells, macrophages, T‐cells, and B‐cells was detected in sarcoid granulomas [116]. Interestingly, it was also found to be upregulated in alveolar macrophages in a neutrophilic asthma model, suggesting its involvement in immune responses in the course of asthma [116]. Efzofitimod, an immune agent specifically binding NPR2, is currently undergoing a multicenter double‐blind placebo‐controlled randomized control trial in pulmonary sarcoidosis patients [116].

In the treatment of severe asthma, several monoclonal antibodies are approved and used in clinical practice, such as anti‐IgE (omalizumab), anti‐IL‐5 (benralizumab and mepolizumab), anti‐TSLP (tezepelumab), and both anti‐IL‐4 and anti‐IL‐13 (dupilumab) [116, 118–120]. Although eosinophilic airway inflammation in type 2 asthma is well recognized and treated, no effective biologic treatment is available in non‐type 2 or neutrophilic asthma [116, 121]. Etiopathogenesis and biomarkers of non‐type 2 asthma are poorly understood and require further research [116]. Several neutrophilic inflammation targets are currently investigated, including IL‐1, IL‐6, IL‐8, IL‐17, IL‐23, and TNF‐α [116, 122].

Increased levels of TNF‐α have been detected in the airways of patients with severe asthma, during exacerbations and after allergen challenge [123]. In animal models, encouraging evidence emerged regarding anti‐TNF‐α therapy, as infliximab and adalimumab proved to reduce airway hyperreactivity and inflammatory cell infiltration, respectively [123]. In humans, there are reports highlighting the potential of etanercept and infliximab to improve severe asthma, lung function, airway inflammation, and the frequency of exacerbations [123–126]. The evidence suggests that TNF‐α inhibitors may prove particularly effective in the treatment of refractory asthma [123].

Hopefully, the ongoing clinical trials will provide further information on the mechanisms and efficacy of the aforementioned biologic agents in the treatment of refractory asthma and sarcoidosis and, perhaps, target both diseases in the future [117, 121].

## 4. Summary

Currently, it is not possible to confidently confirm or exclude asthma in patients with pulmonary sarcoidosis, who present bronchospastic symptoms and BHR (or otherwise confirmed variable obturation). Due to the suspected high prevalence of BHR in both asthma and sarcoidosis, a positive bronchial challenge test does not conclusively determine the diagnosis of concurrent asthma in symptomatic individuals, which should be made only after careful and prolonged observation. Diagnostic challenges often lead to either overdiagnosis of asthma in patients with confirmed sarcoidosis or withholding appropriate treatment. Caution is warranted as patients incorrectly diagnosed with asthma are exposed to unnecessary medical expenses and lifelong therapy. Nevertheless, given the beneficial effects of ICS and bronchodilators on symptom control in sarcoidosis, such treatment may be temporarily considered if it improves the quality of patients’ life and potentially allows discontinuation of OCS, regardless of asthma status.

Due to the scarcity of clinical trials and diagnostic guidelines for treating patients with coexisting asthma and sarcoidosis, as well as sarcoidosis with BHR, clinicians still have to rely on their own experience and follow‐up observations. Further research is needed regarding the prevalence of BHR in patients with asthma and sarcoidosis, taking into account its impact on the quality of the patients’ life. Finally, the inflammatory mediation regarding pulmonary fibrosis focused on TGF‐β, Th1/Th2 balance in sarcoidosis, as well as BHR mechanisms responsible for the relationship between asthma and sarcoidosis require further investigation in an attempt to address the issues encountered in clinical practice.

## Author Contributions

Agata Anna Lewandowska, Dorota Waśniowska, and Cezary Rybacki conceived the concept of the article and formulated main conclusions based on available data. Helena Mirus‐Arabik created figures presented in the article. Małgorzata Kołodziej, Aleksandra Gaczkowska, Ola Duszyńska, Michał Graczyk, and Krzysztof Bułat participated in data collection and analysis.

## Funding

No funding was received for this manuscript.

## Ethics Statement

The authors have nothing to report.

## Conflicts of Interest

The authors declare no conflicts of interest.

## Data Availability

Data sharing is not applicable to this article as no datasets were generated or analyzed during the current study.
